# Liftoff: The Blossoming of Contraceptive Implant Use in Africa

**DOI:** 10.9745/GHSP-D-17-00396

**Published:** 2018-03-21

**Authors:** Roy Jacobstein

**Affiliations:** a IntraHealth International, Chapel Hill, NC, USA.

## Abstract

This article draws from national surveys of every sub-Saharan African country with at least 1 recent survey published between 2015 and 2017 and 2 prior surveys from 2003 to 2014. Twelve countries comprising over 60% of the region's population met these inclusion criteria. The analysis considers recent and longer-term changes in 3 key variables: modern contraceptive prevalence rate (mCPR), method-specific prevalence, and a method's share of the current modern method mix. As recently as 2011, implant CPR in sub-Saharan Africa was only 1.1%. Since then, sizeable price reductions, much-increased commodity supply, greater government commitment to rights-based family planning, broader WHO eligibility guidance, and wider adoption of high-impact service delivery practices have resulted in expanded client access and marked increases in implant prevalence and share of the method mix. Ten of the 12 countries now have an implant CPR around 6% or higher, with 3 countries above 11%. Increased implant use has been the main driver of the increased mCPR attained by 11 countries, with gains in implant use alone exceeding combined gains in use of injectables, pills, and intrauterine devices. In countries as diverse as Burkina Faso and Ethiopia, Democratic Republic of the Congo and Ghana, Kenya and Senegal, implant use now accounts for one-fourth to one-half of all modern method use. Implants have become the first or second most widely used method in 10 countries. In the 7 countries with multiple surveys conducted over a 2- to 3-year span between 2013–14 and 2016–17, average annual gains in implant prevalence range from 0.97 to 4.15 percentage points; this contrasts to historical annual gains in use of all modern methods of 0.70 percentage points in 42 sub-Saharan African countries from 1986 to 2008. Implant use has risen substantially and fairly equitably across almost all sociodemographic categories, including unmarried women, women of lower and higher parity, women in all 5 wealth quintiles, younger and older women, and women residing in rural areas. A notable exception is the category of nulliparous married women, whose implant use is mostly below 1%. These attainments represent a major success story not often seen in family planning programming. With continued program commitment and donor support, these trends in implant uptake and popularity are likely to continue for the next few years. This implies even greater need for the international family planning community to maintain its focus on rights-based programming, ensuring reliable access to implant removal as well as insertion services, and addressing issues of financing and sustainability.

## Abstract (precis)

Contraceptive implant use is rising rapidly, substantially, and equitably in many sub-Saharan African countries, across almost all sociodemographic categories. Gains in implant use have exceeded combined gains for IUDs, pills, and injectables. Key contributing factors include sizeable reductions in commodity cost, much-increased commodity supply, greater government commitment to expanded method choice, and wider adoption of high-impact service delivery practices that broaden access and better reach underserved populations. Continued progress in meeting women's reproductive intentions with implants calls for further investment in quality services for both insertion and removal, and for addressing issues of financing and sustainability.

## INTRODUCTION

This programmatic review and analysis highlights the marked uptake of contraceptive implants that has been occurring in much of sub-Saharan Africa over the past several years. Although implants have many attractive features, including convenience, very high effectiveness, and long duration of action, they had been marginal methods for many years in family planning programs, largely because of high commodity cost—once upwards of US$20/set. As recently as 2011, the contraceptive prevalence rate (CPR) for implants in sub-Saharan Africa was only 1.1% among married women, including 0.6% in Western Africa, 0.3% in Middle Africa, and 0.1% in Southern Africa.^1^ Use of implants among sexually active unmarried women was likely even lower. In the subsequent few years, however, the situation has been changing greatly for implants in terms of overall use; use by women in almost all sociodemographic categories; share of the method mix; and contribution to countries' gains in the modern contraceptive prevalence rate (mCPR) and achievement of Family Planning 2020 (FP2020) goals. This article analyzes these changes in 12 countries that comprise 61% of sub-Saharan Africa's population of 1.03 billion people,^2^ noting important achievements and trends and assessing to what extent the “immense potential of wider implant availability”^3^ is being realized.

As recently as 2011, prevalence of implant use was only 1.1% in sub-Saharan Africa.

## METHODS

### Data Sources

This article draws from 2 sources of representative national population data: Demographic and Health Surveys (DHS) and Performance Monitoring and Accountability 2020 (PMA2020) surveys.^4^^,^^5^ It analyzes recent changes in contraceptive use and method mix among married women and sexually active unmarried women in every sub-Saharan African country meeting 3 inclusion criteria:

1. At least 1 DHS or PMA2020 survey was conducted in the country between 2015 and 2017
2. Information from this latest survey was available online in a DHS Final Report or PMA2020 Family Planning Brief (as of December 31, 2017)
3. At least 2 previous DHS surveys were conducted in the previous decade (between 2003 and 2014).

Twelve countries—Burkina Faso, Democratic Republic of the Congo (DRC), Ethiopia, Ghana, Kenya, Malawi, Niger, Nigeria, Senegal, Tanzania, Uganda, and Zimbabwe—met these inclusion criteria and are included in the analysis. The PMA2020 survey for DRC pertains only to its capital city, Kinshasa (although DRC/Kinshasa will be considered a “country” in terms of this article). Several countries with active family planning programs, including Madagascar, Mozambique, Rwanda, South Africa, and Zambia, did not meet inclusion criteria as their latest surveys were not conducted recently or often enough to reflect a rapidly evolving situation.

Assessment of comparability of DHS and PMA2020 data is beyond the scope of this article. However, in 3 of the 4 countries with DHS and PMA2020 surveys conducted the same year, Ghana (2014), DRC/Kinshasa (2014), and Ethiopia (2016), mCPR and implant CPR figures were comparable. In the fourth country, Kenya (2014), the figures were almost identical: mCPR, 53.2% (DHS) and 53.4% (PMA2020); implant CPR, 9.9% (DHS) and 9.8% (PMA2020). For purposes of this analysis, the survey types are treated as comparable.

To further contextualize the dynamics of implant uptake in the 12 countries under review, approximately 75 additional DHS surveys conducted from 1986 onward in over 25 countries in all regions were examined for levels and rates of increase in overall mCPR and individual modern method CPR (pills, injectables, intrauterine devices [IUDs], female sterilization). The analysis also draws on service statistics supplied by 2 international NGOs active in family planning service delivery, Marie Stopes International (MSI) and Population Services International (PSI). These data include provision of implants in sub-Saharan Africa by MSI from 2008 to 2017, and provision of implants and IUDs by PSI from 2013 to 2017. Finally, the analysis cites data from the Reproductive Health Interchange maintained by the United Nations Population Fund (UNFPA; https://www.unfpaprocurement.org/rhi-home) to quantify the number of implants procured and supplied to sub-Saharan African countries between 2013 and 2017.

### Variables

Three key family planning variables are analyzed across the past decade-plus (2003–2017) among both married women of reproductive age (defined as both currently married women and women living in a consensual union) and unmarried sexually active women of reproductive age:

1. **mCPR:** The percentage of women currently using any modern method of contraception (male or female sterilization, IUDs, oral contraceptive pills, injectables, implants, male or female condoms, diaphragm/foam/jelly, the Lactational Amenorrhea Method, the Standard Days Method, or “other” modern methods such as the cervical cap or contraceptive sponge).
2. **Method-specific CPR:** The percentage of women currently using the specific method in question; for example, implant CPR refers to the percentage of women currently using the implant, and is sometimes referred to as “implant prevalence” or “prevalence of implant use” in this article.
3. **A method's share of the current modern contraceptive method mix:** The percentage of current modern method users who use the particular method in question, providing a profile of the relative level of use of that particular method; for example, the implant's share of the current modern method mix provides a depiction of how widely used the implant is among modern method users. (Note the denominator for this variable is modern method users, whereas the denominator for the previous 2 variables is all women. As users of traditional methods and nonusers of contraception are removed from the denominator, the percentage share of the remaining methods rises in comparison with the method's CPR. In this article, “method mix” or “current method mix” refers to the current modern method mix.)

Unless otherwise indicated, prevalence figures for any of these 3 variables refer to those for married women.

### Analysis

The analysis starts with consideration of changes over the past decade-plus (2003–2017) in the mCPR, implant CPR, and implant share of the method mix for both married and sexually active unmarried women across the 12 included countries. Three data points are considered for each country, from an “early,” “middle,” and latest available survey. Implant prevalence is then further analyzed according to the key sociodemographic categories of parity, age, residence, and wealth quintile for the 7 countries with recent/latest DHS surveys conducted between 2014 and 2016. (PMA2020 Family Planning Briefs afford more recent data, from 2016 to 2017, which is very useful in fast-changing situations as is the case with implant uptake. However, these briefs do not provide method-specific data disaggregated by sociodemographic categories other than marital status.) For this article, PMA2020 provided additional data on the implant's share of the method mix, disaggregated by age, parity, residence, and wealth.) Next, changes over the past decade-plus in implant prevalence and share of the current method mix in all 12 countries are compared with those for the other LARC method, the IUD, and the other commonly used hormonal methods, pills and injectables. Finally, longer-term (2008–2017) and very recent (2013–14 to 2016–17) trends in mCPR and method-specific CPR are analyzed in terms of total and annual percentage-point gains in prevalence.

## FINDINGS

### Substantial Uptake by Married Women

#### High Implant CPR

**FIGURE 1.**
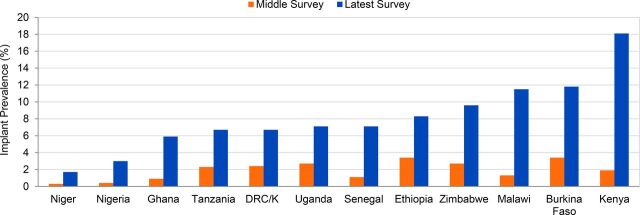
Marked Increases in Implant Use by Married Women, 2008–14 to 2015–17 Abbreviations: DHS, Demographic and Health Survey; DRC/K, Democratic Republic of the Congo/Kinshasa only; PMA2020, Performance Monitoring and Accountability 2020. Data sources: For earlier year (middle survey), DHS surveys for each country; for later year (latest survey), most recent DHS or PMA2020 survey as of December 31, 2017, as indicated in Table 1. Left-hand and right-hand bars for each country correspond respectively to their middle- and upper-row values in Table 1, Column 3.

Implant CPR was very low a decade ago or earlier in the 12 countries under review: 0.6% or lower in 8 countries (including those with data “not available”), and only 1.0% to 1.7% in the other 4 countries (Table 1, Column 3, lowermost rows). Now, among a larger population base of married women, 9 of these 12 countries have an implant CPR of almost 7% or higher (Figure 1). Three countries—Burkina Faso, Kenya, and Malawi—have an implant CPR above 11%. The only countries with an implant CPR below 5.9% are Nigeria (3.0%), whose Kaduna and Nasarawa states have implant CPRs above 6%, and Niger (1.7%), whose capital, Niamey, has an implant CPR of 8.0%. Low-mCPR countries of francophone West and Central Africa (Burkina Faso, DRC/Kinshasa, Senegal) as well as high-mCPR, anglophone countries of Eastern and Southern Africa (Kenya, Malawi, Zimbabwe) have attained high levels of implant use, as have Ethiopia, Ghana, Tanzania, and Uganda. Nearly 1 in every 5 married women in Kenya uses an implant, as does 1 in every 8 to 9 married women in Burkina Faso and Malawi. Kenya's 18.1% prevalence of implant use is the highest in the world.

**TABLE 1.** Trends in the mCPR, Implant CPR, and Implant Share of Current Method Mix Among Married Women and Sexually Active Unmarried Women, 2003–2017

Column 1	Married Women			Sexually Active Unmarried Women		
	Column 2	Column 3	Column 4	Column 5	Column 6	Column 7
Country and Data Source	mCPR	Implant CPR	Implant Share of Method Mix	mCPR	Implant CPR	Implant Share of Method Mix
**Kenya PMA R5 2016**	**59.9**	**18.1**	**30.2**	**53.7**	**8.1**	**15.0**
Kenya DHS 2008-09	39.4	1.9	4.8	45.1	1.2	2.7
Kenya DHS 2003	31.5	1.7	5.4	44.3	2.0	4.5
**Burkina Faso PMA R4 2016**	**24.6**	**11.8**	**48.1**	**38.8**	**7.1**	**18.3**
Burkina Faso DHS 2010	15.0	3.4	22.7	58.7	2.3	3.9
Burkina Faso DHS 2003	8.6	1.2	14.0	55.7	0.9	1.6
**Malawi DHS 2015–16**	**58.1**	**11.5**	**19.8**	**43.2**	**5.8**	**13.4**
Malawi DHS 2010	42.2	1.3	3.1	46.3	0.9	1.9
Malawi DHS 2004	28.1	0.5	1.8	24.3	0.0	0.0
**Zimbabwe DHS 2015**	**65.8**	**9.6**	**14.6**	**66.4**	**14.4**	**21.7**
Zimbabwe DHS 2010–11	57.3	2.7	4.7	61.5	2.7	4.4
Zimbabwe DHS 2005-06	58.4	1.2	2.1	60.2	0.0	0.0
**Ethiopia PMA R5 2017**	**35.2**	**8.3**	**23.7**	**47.1**	**15.6**	**33.2**
Ethiopia DHS 2011	27.3	3.4	12.5	52.3	2.4	4.6
Ethiopia DHS 2005	13.9	0.2	1.4	43.3	0.0	0.0
**Senegal DHS 2016**	**23.1**	**7.1**	**30.7**	**47.9**	**5.9**	**12.3**
Senegal DHS 2010–11	12.1	1.1	9.1	25.6	3.1	12.4
Senegal DHS 2005	10.3	0.6	5.8	43.3	0.6	5.8
**Uganda PMA R5 2017**	**33.9**	**7.1**	**20.8**	**45.5**	**4.0**	**8.7**
Uganda DHS 2011	26.0	2.7	10.4	44.3	2.4	5.4
Uganda DHS 2006	17.9	0.3	1.7	46.9	0.0	0.0
**DRC/K PMA R5 2016**	**23.4**	**6.7**	**28.6**	**41.8**	**3.5**	**8.3**
DRC/K DHS 2013–14	19.0	2.4	12.6	NA^b^	NA^b^	NA
DRC/K DHS 2007	14.1	NA^a^	NA^a^	NA^b^	NA^b^	NA
**Tanzania DHS 2015–16**	**32.0**	**6.7**	**20.9**	**45.8**	**7.7**	**16.8**
Tanzania DHS 2010	27.4	2.3	8.4	44.7	2.8	6.3
Tanzania DHS 2004–05	20.0	0.5	2.5	35.7	0.5	1.4
**Ghana PMA R5 2016**	**25.8**	**5.9**	**23.0**	**37.6**	**7.8**	**20.8**
Ghana DHS 2008	16.6	0.9	5.4	33.8	0.8	2.4
Ghana DHS 2003	18.7	1.0	5.3	31.6	0.3	0.9
**Nigeria PMA R2 2017**	**16.1**	**3.0**	**18.8**	**34.9**	**0.8**	**2.2**
Nigeria DHS 2013	9.8	0.4	4.1	54.9	0.4	0.7
Nigeria DHS 2008	9.7	0.0	0.0	42.4	0.1	0.2
**Niger PMA R1 2016**	**14.4**	**1.7**	**11.9**	**NA** ^b^	**NA** ^b^	**NA** ^b^
Niger DHS 2012	12.2	0.3	2.5	39.9	0.0	0.0
Niger DHS 2006	5.0	NA^a^	NA^a^	NA^b^	NA^b^	NA^b^

Abbreviations: CPR, contraceptive prevalence rate; DHS, Demographic and Health Survey; DRC/K, Democratic Republic of the Congo/Kinshasa only; mCPR, modern contraceptive prevalence rate; NA, not available; PMA, Performance Monitoring and Accountability 2020; R, round.

Notes: Uppermost entry for each country, shown in boldface, is the latest available DHS survey report or PMA2020 Family Planning Brief as of December 31, 2017. Table ordered according to implant CPR for married women (Column 3). All data reported as percentages.

a Implants included in “other modern methods” category.

b Data not provided in survey report.

Implant CPR among married women is now almost 7% or higher in 9 of 12 study countries.

Kenya has attained an implant CPR of 18.1%, the highest in the world.

#### High Share of the Method Mix

**FIGURE 2.**
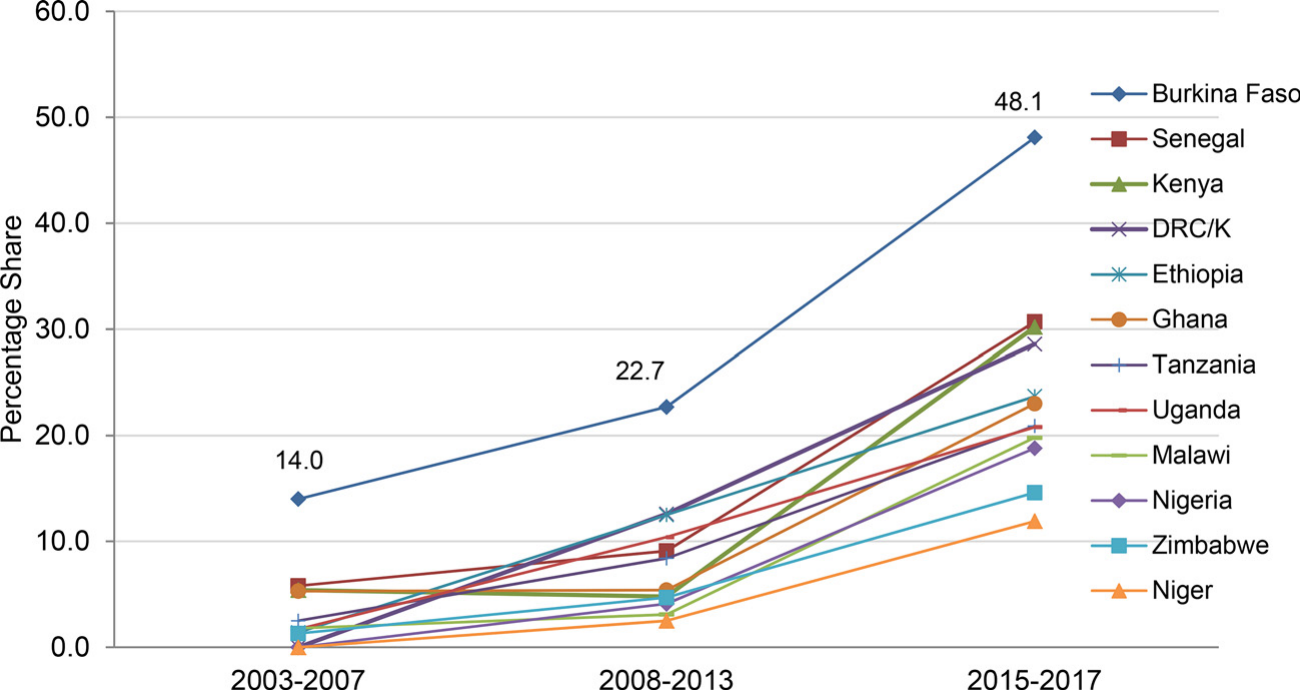
Implants Have Markedly Rising Share of Method Mix Among Married Women, 2003–2017 Abbreviations: DHS, Demographic and Health Survey; DRC/K, Democratic Republic of the Congo/Kinshasa only; PMA2020, Performance Monitoring and Accountability. Data sources: For 2003–07 and 2008–13, DHS surveys for each country; for 2015–17, most recent DHS or PMA2020 survey as of December 31, 2017, as indicated in Table 1.

Implants' share of the current modern method mix is higher than implant CPR in numerical terms (as non-users of modern contraception drop out of the denominator). Implants now account for almost 20% or more of all current modern method use in 10 of the countries under review (Table 1, Column 4). In contrast, a decade ago the implant was little-used, constituting less than 6% of the method mix in all 12 countries except Burkina Faso (where it was 14%), and around 2% or less in 6 countries (including Niger and DRC/Kinshasa, where implant use was so low it was included only in the “other modern methods” category) (Figure 2). In both the high-prevalence milieu of Kenya and the lower-prevalence milieu of Senegal, almost 1 in every 3 married contraceptive users now relies on an implant, as do 1 in every 4 to 5 married contraceptive users in DRC/Kinshasa, Ethiopia, Ghana, Malawi, Nigeria, Tanzania, and Uganda. In Burkina Faso, implants are the most widely used method, accounting for nearly half (48.1%) of all modern method use. In 9 of the other 11 countries, implants have become the second most widely used of all modern methods by married women. (The exceptions are Niger and Nigeria, where implants are the third most widely used modern method.)

Implants are the 1st or 2nd most widely used method by married women in 10 diverse sub-Saharan African countries.

### Comparably High Rates of Implant Use by Unmarried Women

**FIGURE 3.**
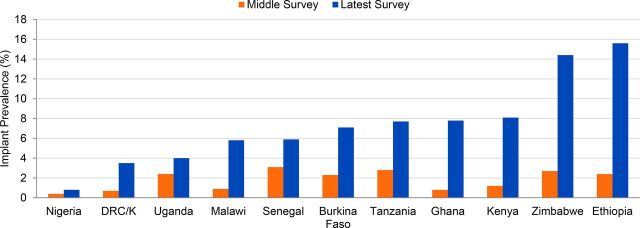
Comparable Increases in Implant Use by Sexually Active Unmarried Women, 2008–14 to 2015–17 Abbreviations: DHS, Demographic and Health Survey; DRC/K, Democratic Republic of the Congo/Kinshasa only; PMA2020, Performance Monitoring and Accountability 2020. Note: Niger's survey reports did not include data on specific method use by unmarried women, and thus are not included in this figure. Data sources: For earlier year (middle survey), DHS surveys for each country; for later year (latest survey), most recent DHS or PMA2020 survey as of December 31, 2017, as indicated in Table 1. Left-hand and right-hand bars for each country correspond to their middle- and upper-row values in Table 1, Column 6.

Sexually active unmarried women are using contraceptive implants at comparably high levels as married women (Table 1, Columns 6 and 7). Implant prevalence among sexually active unmarried women ranges from around 6% to over 15% in 8 of the 11 countries providing data on such use (Figure 3). This contrasts greatly to the situation of a decade earlier, when only 2 countries had an implant prevalence above 0.6%, and 6 countries either had an implant prevalence of 0.0% or did not even list implants as a specific method in their survey reports. The prevalence of implant use by sexually active unmarried women *exceeds* that of married women in Ethiopia, Ghana, Tanzania, and Zimbabwe. One in every 7 sexually active unmarried women in Ethiopia and Zimbabwe uses an implant, as does nearly 1 in every 12 in Ghana, Kenya, and Tanzania. In the socioculturally conservative contexts of francophone West Africa, sexually active unmarried women have attained an implant prevalence of 5.9% in Senegal and 7.1% in Burkina Faso. Implant use accounts for one-third (33.2%) of all modern method use by sexually active unmarried women in Ethiopia, and 15% to 22% of their use in Burkina Faso, Ghana, Kenya, Tanzania, and Zimbabwe. These attainments are especially noteworthy because unmarried women generally encounter greater barriers to family planning access and use than do married women,^6^^,^^7^ particularly in accessing provider-dependent methods like implants.

Sexually active unmarried women have comparably high levels of implant use.

### Substantial Uptake in Almost All Sociodemographic Categories

Women in almost all sociodemographic categories are accessing implants in substantial and generally equitable proportions. This can be readily seen in Table 2, for married women in the 7 countries with a recent DHS survey between 2014 and 2016, in the sociodemographic categories of parity 1 or higher, age above 19, all 5 wealth quintiles, and place of residence. The category of married women ages 15–19, which includes both parous and nulliparous women (women of parity 0, i.e., without children), has noticeably lower implant CPR levels, about half as high as levels for women in older age brackets in 6 of the 7 countries. The category of nulliparous married women (of any age) stands in further distinct contrast, as seen in Table 3. In 5 of the 7 countries (Kenya, Malawi, Senegal, Tanzania, and Zimbabwe), the implant CPRs of nulliparous married women range only from 0.3% to 0.7%, levels many orders of magnitude lower than implant CPRs of women at higher parities. In the other 2 countries, Ethiopia and Ghana, nulliparous women have higher levels of implant uptake, 4.7% and 4.4%, respectively, although these levels are generally lower than for parous women in those countries.

**TABLE 2.** Substantial and Generally Equitable Use of Implants in Almost All Sociodemographic Categories, Married Women, 7 Countries With Recent DHS Surveys

Implant CPR	Ethiopia DHS 2016	Ghana DHS 2014	Kenya DHS 2014	Malawi DHS 2015–16	Senegal DHS 2016	Tanzania DHS 2015–16	Zimbabwe DHS 2015
**All married women**	**7.9**	**5.2**	**9.9**	**11.5**	**7.1**	**6.7**	**9.6**
**Parity**							
0	**4.7**	**4.4**	**0.4**	**0.6**	**0.7**	**0.5**	**0.3**
1–2	10.5	4.0	10.8	13.3	6.7	7.6	8.3
3–4	8.8	5.6	11.2	13.1	7.2	7.7	12.0
5+	5.6	6.6	8.9	9.7	10.8	6.5	12.6
**Age group, years**							
15–19	4.9	6.1	5.4	5.1	2.4	2.7	3.6
20–24	8.7	5.0	9.6	12.3	6.5	8.1	9.9
24–29	9.8	7.2	12.9	17.2	6.1	9.2	9.6
30–34	8.4	6.9	11.9	15.2	7.9	8.3	11.7
35–39	8.4	4.1	10.4	10.0	9.8	6.6	10.8
**Residence**							
Urban	11.0	4.6	12.0	12.8	9.1	6.4	12.0
Rural	7.3	5.8	8.6	11.3	5.7	6.9	8.4
**Wealth quintile**							
Lowest	5.0	4.3	5.7	10.4	6.2	4.5	7.3
Second	7.7	3.3	10.1	10.5	7.2	6.8	8.6
Middle	8.7	6.6	9.8	11.7	8.6	7.8	9.0
Fourth	7.9	5.4	11.1	11.4	6.7	8.7	10.7
Highest	9.9	3.8	11.7	13.5	7.1	6.1	12.2

Abbreviations: CPR, contraceptive prevalence rate; DHS, Demographic and Health Survey.

Note: All data reported as percentages.

**TABLE 3.** Low Implant Use by Nulliparous Married Women, Substantial Use by Married Women With Children, 7 Countries With Recent DHS Surveys

Country and Data Source	Implant CPR at Parity 0	Range of Implant CPRs at Parity 1 and Higher
Zimbabwe DHS 2015	0.3	8.3–12.6
Kenya DHS 2014	0.4	8.9–11.2
Tanzania DHS 2015–16	0.5	6.5–7.7
Malawi DHS 2015–16	0.6	9.7–13.3
Senegal DHS 2016	0.7	6.7–10.8
Ghana DHS 2014	4.4	4.0–6.6
Ethiopia DHS 2016	4.7	5.6–10.5

Abbreviations: CPR, contraceptive prevalence rate; DHS, Demographic and Health Survey.

Notes: Table ordered from lowest to highest Implant CPR at parity 0. All data reported as percentages.

Women in almost all sociodemographic categories are accessing implants in substantial and generally equitable proportions.

In additional data provided by PMA2020 for this article (Sally Safi, written communication, February 2018), the marked differences in implant uptake between nulliparous married women and women of higher parity are also seen in the 5 countries that are not included in Table 3. The implant's share of the method mix (not implant CPR) among married women at parity 0 is 0.0% in all 5 countries. This contrasts to method-mix shares among higher parity women ranging from 44.0% to 48.4% in Burkina Faso, 7.1% to 15.8% in DRC/Kinshasa, 7.5% to 20.1% in Niger, 6.3% to 14.1% in Nigeria, and 13.0% to 19.9% in Uganda. The implant's share of the method mix in these countries also exhibits similar patterns across other sociodemographic categories as does implant CPR in the other 7 countries. For example, in Burkina Faso, the implant's share of the method mix ranges from 38.8% to 53.6% among women 20–39 and from 39.4% to 53.2% across wealth tertiles. In Nigeria, the implant's share of the method mix is 3.9% among women 15–19 compared with 8.2% to 15.6% among older women. Similarly, in Niger implants' share of method use among women aged 15–19 (most of whom are married) was 0.7%, whereas it ranged from 6.4% to 15.9% among women in higher age brackets.

### Trend in Use of Other Reversible Methods

#### Use of Injectables Still Rising, but Share of Method Mix Declining

**FIGURE 4.**
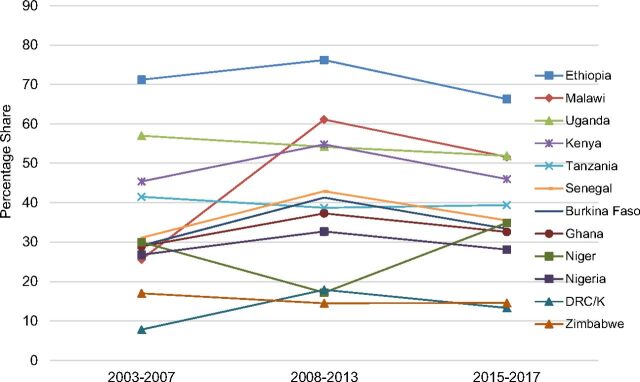
Injectables Share of Method Mix Plateauing or Falling Among Married Women, 2003–2017 Abbreviations: DHS, Demographic and Health Survey; DRC/K, Democratic Republic of the Congo/Kinshasa only; PMA2020, Performance Monitoring and Accountability. Data sources: For 2003–07 and 2008–13, DHS surveys for each country; for 2015–17, most recent DHS or PMA2020 survey, as of December 31, 2017, as indicated in Table 4.

Injectables are widely used in sub-Saharan Africa,^1^ and their use is still rising in 11 of the 12 countries under review (Table 4, Column 5). Injectables prevalence ranges from a low of 3.1% in DRC/Kinshasa to a high of 30.0% in Malawi. In 7 countries, injectables prevalence is around 10% or higher and in 9 countries injectables' share of the method mix is over 30% (Table 4, Column 6). With implants' rising—and faster-rising—CPRs, however, injectables' still-predominant share of the method mix, 28% to 66% in the 9 highest-use countries, has declined in 9 countries and plateaued in 2 others (Figure 4). Three of the 4 countries where injectables comprise over half the modern method mix, Kenya, Ethiopia, and Malawi, have experienced declines of 9 to 10 percentage points in the injectables' share of the method mix during the past 4 to 8 years as implant uptake has risen substantially there.

**TABLE 4.** Method-Specific CPR and Share of Current Modern Method Mix for Implants, Injectables, IUDs, and Pills, Married Women, 2003–2017

Column 1	Column 2	Column 3	Column 4	Column 5	Column 6	Column 7	Column 8
Country and Data Source	mCPR	Implant CPR	Implant Share of Method Mix	Injectables CPR	Injectables Share of Method Mix	IUD CPR/Share of Method Mix	Pill CPR/Share of Method Mix
**Kenya PMA R5 2016**	**59.9**	**18.1**	**30.2**	**27.6**	**46.0**	**3.5/5.9**	**5.1/8.5**
Kenya DHS 2008–09	39.4	1.9	4.8	21.6	54.8	1.6/4.1	7.2/18.3
Kenya DHS 2003	31.5	1.7	5.4	14.3	45.4	2.4/7.6	7.5/23.8
**Burkina Faso PMA R4 2016**	**24.6**	**11.8**	**48.1**	**8.2** ^b^	**33.4** ^b^	**0.9/3.5**	**2.8/11.5**
Burkina Faso DHS 2010	15.0	3.4	22.7	6.2	41.3	0.3/2.0	3.2/21.3
Burkina Faso DHS 2003	8.6	1.2	14.0	2.5	29.1	0.4/4.7	2.2/25.6
**Malawi DHS 2015–16**	**58.1**	**11.5**	**19.8**	**30.0**	**51.6**	**1.1/1.9**	**2.4/4.1**
Malawi DHS 2010	42.2	1.3	3.1	25.8	61.1	0.3/0.7	2.5/5.9
Malawi DHS 2004	28.1	0.5	1.8	18.0	25.6	0.1/0.4	2.0/7.1
**Zimbabwe DHS 2015**	**65.8**	**9.6**	**14.6**	**9.6**	**14.6**	**0.6/1.0**	**41.1/62.5**
Zimbabwe DHS 2010–11	57.3	2.7	4.7	8.3	14.5	0.2/0.3	41.3/72.1
Zimbabwe DHS 2005–06	58.4	1.2	2.1	9.9	17.0	0.3/0.5	43.0/73.6
**Ethiopia PMA R5 2017**	**35.2**	**8.3**	**23.7**	**24.1**	**66.3**	**1.2/3.3**	**1.8/5.2**
Ethiopia DHS 2011	27.3	3.4	12.5	20.8	76.2	0.3/1.1	2.1/7.7
Ethiopia DHS 2005	13.9	0.2	1.4	9.9	71.2	0.2/1.4	3.1/22.3
**Senegal DHS 2016**	**23.1**	**7.1**	**30.7**	**8.2**	**35.5**	**1.6/6.9**	**4.6/20.0**
Senegal DHS 2010–11	12.1	1.1	9.1	5.2	43.0	0.6/5.0	4.1/33.9
Senegal DHS 2005	10.3	0.6	5.8	3.2	31.1	0.5/4.9	3.6/35.0
**Uganda PMA R5 2017**	**33.9**	**7.1**	**20.8**	**17.6** ^b^	**51.9** ^b^	**0.9/2.6**	**2.7/8.1**
Uganda DHS 2011	26.0	2.7	10.4	14.1	54.2	0.5/1.9	2.9/11.2
Uganda DHS 2006	17.9	0.3	1.7	10.2	57.0	0.2/1.1	2.9/16.2
**DRC/K PMA R5 2016**	**23.4**	**6.7**	**28.6**	**3.1** ^b^	**13.3**	**1.0/4.2**	**3.7/15.6**
DRC/K DHS 2013–14	19.0	2.4	12.6	3.4	17.9	0.5/2.6	3.0/15.8
DRC/K DHS 2007	14.1	NA^a^	NA^a^	1.1	7.8	NA^a^	2.2/15.6
**Tanzania DHS 2015-16**	**32.0**	**6.7**	**20.9**	**12.6**	**39.4**	**0.9/2.8**	**5.5/17.2**
Tanzania DHS 2010	27.4	2.3	8.4	10.6	38.7	0.6/2.2	6.7/24.5
Tanzania DHS 2004-05	20.0	0.5	2.5	8.3	41.5	0.2/1.0	5.9/29.5
**Ghana PMA R5 2016**	**25.8**	**5.9**	**23.0**	**8.4**	**32.6**	**0.5/2.0**	**4.5/17.5**
Ghana DHS 2008	16.6	0.9	5.4	6.2	37.3	0.2/1.2	4.7/28.3
Ghana DHS 2003	18.7	1.0	5.3	5.4	28.9	0.9/4.8	5.5/29.4
**Nigeria PMA R2 2017**	**16.1**	**3.0**	**18.8**	**4.5**	**28.1**	**1.0/6.5**	**2.5/15.4**
Nigeria DHS 2013	9.8	0.4	4.1	3.2	32.7	1.1/11.2	1.8/18.4
Nigeria DHS 2008	9.7	0.0	0.0	2.6	26.8	1.0/10.3	1.7/17.5
**Niger PMA R1 2016**	**14.4**	**1.7**	**11.9**	**5.0**	**34.9**	**0.4/2.9**	**6.8/47.0**
Niger DHS 2012	12.2	0.3	2.5	2.1	17.2	0.1/0.8	5.6/45.9
Niger DHS 2006	5.0	NA^a^	NA^a^	1.5	30.0	0.1/2.0	3.0/60.0

Abbreviations: CPR, contraceptive prevalence rate; DHS, Demographic and Health Survey; DRC/K, Democratic Republic of the Congo/Kinshasa only; DMPA, depot medroxyprogesterone acetate; IUD, intrauterine device; mCPR, modern contraceptive prevalence rate; NA, not available; PMA, Performance Monitoring and Accountability 2020; R, round.

Notes: Uppermost entry for each country is latest available DHS survey report or PMA2020 Family Planning Brief as of December 31, 2017. Table ordered according to implant CPR (Column 3). All data reported as percentages.

a Included in “other modern methods” category.

b Sum of the intramuscular DMPA injectable and the subcutaneous injectable Sayana Press.

Use of injectables is still high, and rising, but their share of the method mix has declined in 9 of 12 countries.

#### IUD Use Still Low, but Modest Gains in All 12 Countries

**FIGURE 5.**
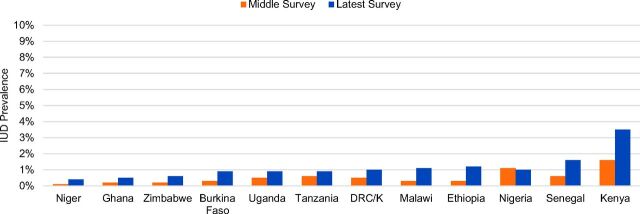
IUD Use Low in All 12 Countries, With Recent Modest Gains, 2008–2017 Abbreviations: DHS, Demographic and Health Survey; DRC/K, Democratic Republic of the Congo/Kinshasa only; PMA2020, Performance Monitoring and Accountability. Data sources: For earlier year (middle survey), DHS surveys for each country; for later year (latest survey), most recent DHS or PMA2020 survey as of December 31, 2017. Left-hand and right-hand bars for each country correspond respectively to middle- and upper-row values in Table 4, Column 7. Data for married women only.

Prevalence patterns for the IUD are very different than for implants (Figure 5). In earlier surveys, 11 of the 12 countries under review had very low levels of IUD prevalence among married women, with most countries' prevalence figures at or below 0.5% and none above 1.6% (Table 4, Column 7). This is consistent with IUD prevalence patterns in sub-Saharan Africa more generally, where IUD CPR among married women is only 0.7%,^1^ and use by sexually active unmarried women has been negligible. More recently, however, modest gains in IUD CPR have been generated in 11 of the 12 countries (Table 5). With only Nigeria's IUD prevalence declining, and by only 0.1 percentage point, gains have ranged from 0.3 percentage points in Ghana, Niger, and Tanzania to 1.9 percentage points in Kenya. Although IUD CPR is still relatively low—1.6% or less in all 12 countries except Kenya, where IUD CPR is 3.5%—it has quadrupled in Ethiopia, Malawi, and Niger, and doubled or tripled in Burkina Faso, Ghana, Kenya, Senegal, and Zimbabwe. IUD use comprises 6.9% of modern method use in Senegal, 6.5% in Nigeria, and almost 6% in Kenya, whose 3.5% IUD prevalence is the highest in Africa. As discussed below, IUDs and implants are often both part of the same LARC-oriented program efforts to broaden a woman's method options by expanding rights-based access to provider-dependent clinical methods.

**TABLE 5.** Comparison of Gains in mCPR and Method-Specific CPR for Implants, Injectables, IUDs, and Pills, Married Women, 2008–2017

Column 1	Column 2	Column 3	Column 4	Column 5	Column 6	Column 7	Column 8	Column 9	Column 10	Column 11
Country and Data Source	mCPR (%)	Total Gain in mCPR (pp)	Implant CPR (%)	Total Gain in Implant CPR (pp)	Injectable CPR (%)	Total Gain in Injectable CPR (pp)	IUD CPR (%)	Total Gain in IUD CPR (pp)	Pill CPR (%)	Total Gain in Pill CPR (pp)
Kenya PMA R5 2016	59.9	**20.5**	18.1	**16.2**	27.6	**6.0**	3.5	**1.9**	5.1	**−2.1**
Kenya DHS 2008–09	39.4		1.9		21.6		1.6		7.2	
Burkina Faso PMA R4 2016	24.6	**9.6**	11.8	**8.4**	8.2^a^	**2.0**	0.9	**0.6**	2.8	**−0.4**
Burkina Faso DHS 2010	15.0		3.4		6.2		0.3		3.2	
Malawi DHS 2015-16	58.1	**15.9**	11.5	**10.2**	30.0	**4.2**	1.1	**0.8**	2.4	**−0.1**
Malawi DHS 2010	42.2		1.3		25.8		0.3		2.5	
Zimbabwe DHS 2015	65.8	**8.5**	9.6	**6.9**	9.6	**1.3**	0.6	**0.4**	40.9	**−0.4**
Zimbabwe DHS 2010–11	57.3		2.7		8.3		0.2		41.3	
Ethiopia PMA R5 2017	35.2	**7.9**	8.3	**4.9**	24.1	**3.3**	1.2	**0.9**	1.8	**−0.3**
Ethiopia DHS 2011	27.3		3.4		20.8		0.3		2.1	
Senegal DHS 2016	23.1	**11.0**	7.1	**6.0**	8.2	**3.0**	1.6	**1.0**	4.6	**0.5**
Senegal DHS 2010–11	12.1		1.1		5.2		0.6		4.1	
Uganda PMA R5 2017	33.9	**7.9**	7.1	**4.4**	17.6 ^a^	**3.5**	0.9	**0.4**	2.7	**−0.2**
Uganda DHS 2011	26.0		2.7		14.1		0.5		2.9	
DRC/K PMA R5 2016	23.4	**4.4**	6.7	**4.3**	3.1 ^a^	**−0.3**	1.0	**0.5**	3.7	**0.7**
DRC/K DHS 2013–14	19.0		2.4		3.4		0.5		3.0	
Tanzania DHS 2015–16	32.0	**4.6**	6.7	**4.4**	12.6	**2.0**	0.9	**0.3**	5.5	**−1.2**
Tanzania DHS 2010	27.4		2.3		10.6		0.6		6.7	
Ghana PMA R5 2016	25.8	**9.2**	5.9	**5.0**	8.4	**2.2**	0.5	**0.3**	4.5	**−0.2**
Ghana DHS 2008	16.6		0.9		6.2		0.2		4.7	
Nigeria PMA R2 2017	16.1	**6.3**	3.0	**2.6**	4.5	**1.3**	1.0	**−0.1**	2.5	**0.7**
Nigeria DHS 2013	9.8		0.4		3.2		1.1		1.8	
Niger PMA R1 2016	14.4	**2.2**	1.7	**1.4**	5.0	**2.9**	0.4	**0.3**	6.8	**1.2**
Niger DHS 2012	12.2		0.3		2.1		0.1		5.6	

Abbreviations: CPR, contraceptive prevalence rate; DHS, Demographic and Health Survey; DMPA, depot medroxyprogesterone acetate; DRC/K, Democratic Republic of the Congo/Kinshasa only; IUD, intrauterine device; PMA, Performance Monitoring and Accountability 2020; pp, percentage point; R, round.

Note: Table ordered according to implant CPR (Column 4).

a Sum of the intramuscular DMPA injectable and the subcutaneous injectable Sayana Press.

With broadened program availability of LARCs, IUD use has risen modestly in 11 of the 12 study countries.

#### Pill Use Below 7%, Share of Method Mix Declining

Pills are generally widely available from multiple private- and public-sector sources. Nonetheless, pill use by married women is low in 11 of the 12 countries under review, excepting Zimbabwe. Prevalence of pill use among married women ranges from 1.8% to 6.8% in the 11 countries, 5 of which have a pill CPR below 2.9% (Table 4, Column 8). Zimbabwe, long a “pill country,” is the notable and striking exception, with a pill CPR of 40.9%. There too, however, implants use has risen 8-fold over the past decade, while pill use has plateaued, declining by several percentage points. Pill CPR has also declined modestly over the past decade in 7 other countries; and gains in pill use in the other 4 countries range only from 0.5 to 0.7 percentage points (Table 5). Pill use by sexually active unmarried women is generally comparable with use by married women (i.e., not considerably higher, as might have been expected). For example, pill CPR among sexually active women is 1.9% in Malawi, 6.4% in Ghana, 6.6% in Kenya (DHS 2014), and 16.0% in Zimbabwe.

### Rapid and Recent Uptake of Implants

The marked increases in implant use described in this analysis are of recent vintage. Although implants had been an approved family planning program method for over 25 years, implant use across these 12 countries between 2003 and 2008 averaged less than 0.7% among married women, and even less among sexually active unmarried women (Table 1, lowermost to middle rows). In the subsequent few years, modest gains arose, with implant CPR averaging 1.9% across the 12 countries. In the past 4 to 8 years, however, implant use has surged in all 12 countries (Table 5, Column 4; Figure 1). Kenya's implant CPR among married women, only 1.9% in 2008–09, quadrupled to 7.4% in 2014 and then more than doubled over the next 2 years, to 18.1% in 2016. Similarly, between 2010 and 2015-16 implant use in Malawi rose 9-fold, from 1.3% to 11.5%, a 785% increase in less than 6 years. In a span of 4 to 8 years, implant use increased 6-fold in Ghana and Niger (from only 0.3%) and approximately 7-fold in Nigeria and Senegal. Comparably rapid and substantial gains in implant access and use have been achieved by sexually active unmarried women, with 6- to 9-fold increases in implant CPR registered in Ethiopia, Ghana, Kenya, Malawi, and Zimbabwe, and a tripling of implant use in Burkina Faso and Tanzania (Figure 3).

Further evidence of the rapidity, recency, scale, and ongoing pace of increased implant uptake is provided in data from the 7 countries with 3 or more serial surveys conducted in a 2- to 3-year span ending in 2016 or 2017 (Table 6). During this very short recent time interval, implant CPRs, which had already been increasing, rose an additional 44% to 145%, more than doubling in DRC/Kinshasa, Ghana, and Uganda. Total gains in implant CPR ranged from lows of 2.3 percentage points in Senegal from 2014 to 2016 to a high of 8.3 percentage points in Kenya from 2014 to 2016. Average annual gains in implant CPR in the 7 countries ranged from 0.97 percentage points (Ethiopia) to 5.35 percentage points (Kenya). In comparison, the average annual gain in use of *all* modern methods (mCPR) in 42 sub-Saharan African countries was only 0.70 percentage points between 1986 and 2008.^8^ The extent and rapid pace of implant uptake is also reflected in the markedly upward slope between midpoint (2008–2013) and endpoint (2015–2017) for the implant's share of the current modern method mix in the 12 countries (Figure 2).

**TABLE 6.** Total and Average Annual Gains in mCPR, Implant CPR, and Implant Share of Method Mix Among Married Women in the 7 Countries With at Least 3 Surveys Between 2013–14 and 2016–17

Column 1	Column 2	Column 3	Column 4	Column 5	Column 6	Column 7	Column 8
Country and Data Source	mCPR (%)	Total Gain in mCPR (pp), First to Latest Survey	Average Annual Gain in mCPR (pp)	Implant CPR (%)	Total Gain in implant CPR (pp), First to Latest Survey	Average Annual Gain in Implant CPR (pp)	Implant's Share of Current Modern Method Mix (%)
**Kenya PMA R5 2016**	**59.9**	**6.5**	**3.25**	**18.1**	**8.3**	**4.15**	**30.2**
Kenya PMA R3 2015	58.8			13.8			23.4
Kenya PMA R1 2014	53.4			9.8			18.3
**Burkina Faso PMA R4 2016**	**24.6**	**6.6**	**3.30**	**11.8**	**3.6**	**1.80**	**48.1**
Burkina Faso PMA R2 2015	20.1			7.9			39.5
Burkina Faso PMA R1 2014	18.0			8.2			45.5
**DRC/K PMA R5 2016**	**23.4**	**4.9**	**1.63**	**6.7**	**5.1**	**1.70**	**28.6**
DRC/K PMA R3 2015	20.4			3.7			18.2
DRC/K PMA R1 2013	18.5			1.6			8.6
**Uganda PMA R5 2017**	**33.9**	**8.3**	**2.77**	**7.1**	**3.8**	**1.27**	**20.8**
Uganda PMA R3 2015	30.0			4.9			16.4
Uganda PMA R1 2014	25.6			3.3			12.8
**Senegal DHS 2016**	**23.1**	**2.8**	**1.40**	**7.1**	**2.3**	**1.15**	**30.7**
Senegal DHS 2015	21.2			5.2			24.5
Senegal DHS 2014	20.3			4.8			23.6
**Ghana PMA R5 2016**	**25.8**	**7.4**	**2.47**	**5.9**	**3.0**	**1.00**	**22.9**
Ghana PMA R3 2014	21.4			3.7			17.4
Ghana PMA R1 2013	18.4			2.9			15.8
**Ethiopia PMA R5 2017**	**35.2**	**1.4**	**0.47**	**8.3**	**2.9**	**0.97**	**23.7**
Ethiopia PMA R3 2015	35.8			7.5			20.9
Ethiopia PMA R1 2014	33.8			5.4			16.0

Abbreviations: CPR, contraceptive prevalence rate; DHS, Demographic and Health Survey; DRC/K, Democratic Republic of the Congo/Kinshasa only; mCPR, modern contraceptive prevalence rate; PMA, Performance Monitoring and Accountability 2020; pp, percentage point; R, round.

Notes: Upper entries are latest surveys available online as of December 31, 2017. Table ordered according to average annual gain in implant CPR (Column 7).

Increases in implant use have been very rapid and very substantial.

### Rising Implant Use the Main Driver of mCPR Gains in 11 of 12 Countries

Whether countries have high, medium, or low mCPR, gains in implant CPR are the predominant driver of the total mCPR gains they have generated over the past 4 to 8 years, in every country except Niger (Table 5). This finding holds for countries that have achieved large average annual gains in mCPR of over 2 percentage points per year (Kenya, Malawi, Senegal, Zimbabwe), as well as countries that have achieved lower but still substantial gains, ranging from 1.15 to 1.76 percentage points per year (Burkina Faso, DRC/Kinshasa, Ethiopia, Ghana, Nigeria, Uganda). Total gains in implant use exceed combined total gains in use of injectables, pills, and IUDs in every country except Niger. Average annual gains in implant CPR in the 7 countries with 3 or more very recent surveys amounted to 44% to 206% of their average annual gains in mCPR (Table 6). Ethiopia's average annual gain in implant CPR from 2014 to 2017 was twice its (modest) mCPR gain (0.97 percentage points vs. 0.47 percentage points, respectively). Kenya's remarkably high average annual gain in implant CPR of 4.15 percentage points also exceeded its substantial average annual gain in mCPR (of 3.25 percentage points). The other 5 countries registered recent average annual gains in implant prevalence that ranged from 1.0 to 1.8 percentage points.

Gains in implant CPR have been the main contributor to mCPR gains in 11 of 12 countries.

### Method-Specific Gain in Use Having Marked Effect on mCPR Uncommon

Large, rapid method-specific gains with substantial effects on mCPR have not often been seen for other modern methods during the past decade of family planning programming. When this phenomenon did occur in earlier years, the method was rapidly becoming or had already become the country's predominant method. In the Latin America and the Caribbean region, increases in female sterilization prevalence constituted sizeable proportions of mCPR gains in Colombia and the Dominican Republic. In Colombia (2000 to 2010), average annual gains in female sterilization CPR and mCPR were 0.78 and 0.89 percentage points, respectively. In the Dominican Republic (1986 to 2007), average annual gains were 0.69 percentage points for female sterilization and 0.95 percentage points for mCPR.

Large, method-specific gains in use have been uncommon in family planning programming.

Several different methods predominate and increased rapidly in Asia. In India, female sterilization CPR gained 0.62 percentage points, and mCPR 0.98 percentage points, annually from 1992–93 to 2005–06. Annual pill use and mCPR in Bangladesh gained 0.75 and 1.06 percentage points, respectively (1993 to 2000). More recently (2000–2014), pill use has also driven increased mCPR in Cambodia, with average annual gains of 0.94 and 1.45 percentage points, respectively. Injectables had similar gains in Indonesia and Nepal. In Indonesia, the average gain in injectables prevalence was 1.27 percentage points (1994–2007); however, the mCPR rose only 2.7 percentage points during that time, implying that the method-specific gain from injectables largely represents substitution effects. Nepal gained 3.5 percentage points in mCPR per year between 1996 and 2001, with injectables and female sterilization each increasing about 1 percentage point per year; however, all 3 measures declined over the subsequent 15 years.

Sizeable gains in injectables use, comparable with those for implants, have also driven sizeable mCPR gains in Africa in the past. In post-genocide Rwanda, mCPR rose from 10.3% in 2005 to 45.1% in 2010, a total gain of 34.8 percentage points and an average annual gain of 7.0 percentage points—the largest such gains ever generated over such a short time in family planning programs. Contributing substantially to those gains, injectables use rose by 21.6 percentage points, an average annual gain of 4.3 percentage points. Injectables use and mCPR also rose substantially in Zambia, by 1.66 and 1.88 percentage points per year from 2007 to 2013–14. Similar gains have occurred in the 12 countries under review. For example, average annual gains in mCPR and injectables prevalence in Ethiopia (2005 to 2011) were 2.23 and 1.67 percentage points, respectively, and in Kenya (2003 to 2008–09), 1.44 and 1.33 percentage points, respectively. Increased injectables use in Malawi (2004 to 2010) accounted for 55% of its sizeable average annual gain in mCPR of 2.35 percentage points. Smaller gains in injectables prevalence during the earlier periods equaled or exceeded mCPR gains in Nigeria and Senegal.

## DISCUSSION

### Why Has Uptake of Implants Been So Rapid and Substantial?

A number of factors have contributed to the increases in implant use documented in this article. Among the most salient are: (1) implants' many positive method characteristics; (2) revised expert guidance supportive of wider client eligibility to receive an implant; (3) greater country commitment to ensuring broad access to a wider choice of methods, including implants; (4) donor and manufacturer action to ensure much-lowered commodity cost and greater commodity availability; and (5) continued and wider reliance on high-impact service delivery practices that expand access and reach underserved populations. These factors are considered in turn.

#### 1. Implants Have Many Positive Method Characteristics

Implants have many positive characteristics that contribute to their rapidly rising popularity:

- **Ease of provision:** Implants can be quickly, safely, and easily inserted in 2–3 minutes by trained providers, including frontline and community workers.^9^
- **Convenience and duration of action:** Whichever implant a client chooses, she can be assured with one action of highly effective contraception for up to 5 years, according to the latest recommendations and studies from the World Health Organization (WHO).^10^
- **Effectiveness:** Implants have the highest effectiveness of all methods, with 1-year failure rates well below 1% in typical use; in comparison, typical-use failure rates of the injectable and pill are 6% and 9%, respectively.^11^
- **Uncomplicated provision:** Implants do not entail pelvic examination or abdominal surgery (like IUDs and female sterilization), generally a positive feature for clients.
- **Ready reversibility:** No further routine action is needed until the client wants the implant removed. (Removal is usually a quick and uncomplicated procedure taking 3–7 minutes; however some removals can be difficult, possibly requiring referral.)
- **Prompt return to fertility:** Prompt return to (former levels of) fertility is a welcome characteristic for women wanting to delay a first birth or space a next birth.
- **Suitable for all reproductive intentions:** In addition to being appropriate for delaying a first birth or spacing a next birth, implants are also appropriate for limiting further births.
- **High client satisfaction/high continuation:** Because of the aformentioned features, implants generally have high client satisfaction, as implied in their high continuation rates, ranging from 78% to 96% at 1 year to 50% to 86% at 3 years.^12^
- **Less demanding on health system infrastructure:** From a programmatic standpoint, implants provision requires less health system infrastructure and less-highly trained staff than other provider-dependent clinical methods.

Implants have many positive method characteristics, including convenience, long duration of action, very high effectiveness, ease of provision, and prompt return to fertility upon removal.

Implants are suitable for all reproductive intentions.

#### 2. Revised Service Delivery Guidance Has Widened Client Eligibility

Guidance from international normative bodies has recently been broadened regarding who can use implants and when use can be initiated. According to WHO almost all women are eligible to use an implant, at any time.^13^ Women can now use implants immediately postpartum, whether or not they are breastfeeding. Nulliparous women can also use implants, as can adolescents and young women, irrespective of age or marital status. In support of this guidance and to further its incorporation into national guidelines and standards of practice, 53 organizations involved in international family planning/reproductive health policy, training, and/or service delivery—including the International Confederation of Midwives (ICM), the International Federation of Gynecology and Obstetrics (FIGO), and the International Planned Parenthood Federation (IPPF)—endorsed a 2015 Global Consensus Statement on the importance of expanding contraceptive choice for adolescents and youth to include long-acting reversible contraception.^14^ In 2016, the American College of Obstetricians and Gynecologists (also a consensus statement endorsee) reconfirmed its 2012 recommendation that its members “encourage adolescents age 15–19 to consider implants and IUDs as the best reversible methods for preventing unintended pregnancy, rapid repeat pregnancy, and abortion.”^15^

Almost all women can use implants at almost all times, including adolescents, nulliparous women, unmarried women, and breastfeeding women.

#### 3. Greater Country Commitments Have Been Made to Ensure Wider Family Planning Access and Method Choice

The landmark July 2012 London Summit on Family Planning, which led to establishment of the global FP2020 development partnership, revitalized national and international attention to family planning.^16^ At that time, 20 national governments as well as donor, civil society, and implementing partner organizations reaffirmed the important socioeconomic, health, and human rights rationales for supporting universal access to family planning. They committed to addressing policy, financing, and service delivery barriers, in order to enable an additional 120 million women and girls to select the contraceptive method of their choice from a broadened range of modern methods, including implants. According to the latest FP2020 annual report, as of July 2017, 41 national governments have made explicit FP2020 commitments to increase funding and prioritization for family planning, and more than 38 million additional clients have accessed family planning services in poor countries, including 16 million women and girls in sub-Saharan Africa.^17^

Greater commitment for family planning and widened method choice, including implants, has been galvanized via the FP2020 global partnership.

Countries have also focused on implants and/or LARCs in promulgating and following their Costed Implementation Plans (CIPs), projecting and planning for markedly increased implant uptake. For example, Ethiopia's 2015–2020 CIP plans for the number of implant users to risefrom 1.7 million women (of 6.7 million family planning users) in 2015 to 3.2 million women (of 9.9 million family planning users) in 2020.^18^ This projected service increase, well on its way to happening (see point number 5 below), constitutes around 25% of Ethiopia's overall FP2020 goal of serving an additional 6.2 million women and girls by 2020. The Ethiopia CIP also commits to “working to identify alternative and sustainable domestic sources for financing healthcare,” while also recognizing that “heavy reliance on out-of-pocket payments is undesirable, as it can make healthcare inaccessible to vulnerable households.” Similarly, Uganda's 2015–2020 CIP projects and plans for more than a tripling of clients who will be relying on implants, from around 230,000 women in 2015 to over 830,000 in 2020.^19^

#### 4. Substantial Reductions in Commodity Cost and Increases in Commodity Availability Have Occurred

Marked reductions in commodity cost have been a key factor in expanding availability of implants. For several decades after their programmatic introduction in the 1980s, implants' commodity cost was around $20 or more per set. (An IUD, in comparison, costs only about $0.40 in the public sector.^20^) Consequently, as recently as 2011 implant CPR was only 0.5% in all developing regions of the world.^1^ In 2007, however, the Bill & Melinda Gates Foundation supported the introduction into the global market of Sino-implant (II), whose commodity cost was around one-third the prevailing cost of other implants. Subsequently, a major outcome that emerged from the 2012 London Summit was the large-scale collaborative agreement between multiple donors, including the Bill & Melinda Gates Foundation, Norwegian Agency for Development Cooperation, Swedish International Development Cooperation Agency, and Children's Investment Fund Foundation, and the implant manufacturers, Bayer (maker of the 2-rod implant, Jadelle) and Merck (maker of the 1-rod implant, Implanon, and its successor, Implanon NXT).^21^ This led to the launch of the Implant Access Program (IAP) in 2012-13, with halving of implant commodity cost to around $8.50 per set and assurance of much greater production, funding, and availability of implants for the world's poorest countries.^22^ As part of their IAP and FP2020 commitments, Bayer and Merck subsequently committed to maintaining their implant access pricing through 2023.^23^^,^^24^ Ethiopia's 2015–2020 CIP projects an implant commodity cost of $8.93 plus $1.85 for “consumables,” which includes allocation of salaries. Sino-implant (II), now marketed as Levoplant, was prequalified by WHO in June 2017 and has been being supplied to sub-Saharan African and other family planning programs at $7.50 to $8.00 per set.^25^ In February 2018, DKT and Dahua Pharmaceutical announced a partnership to provide Levoplant at $6.90 per set in the 69 FP2020 countries.^26^

Public-private partnerships have led to marked reductions in commodity cost, the *sine qua non* of increased implant availability, access, and use.

Between 2013 and 2017, sub-Saharan Africa was supplied with more than 25.7 million implants from donors, mainly UNFPA and the United States Agency for International Development (USAID).^27^ At a halved commodity cost, this represents a cost savings to donors of upwards of $230 million. Over 72% of this total procurement (some still in the pipeline)—more than 18 million implants—was supplied to the 12 countries under review. This includes 4.1 million implants to Tanzania, 4.0 million to Ethiopia, 2.2 million to Nigeria, 1.8 million to Kenya, 1.5 million to DRC, and 1.3 million to Burkina Faso. Without this substantial commodity supply, these countries would not have been able to attain their considerable gains in implant CPR and mCPR as they progress toward achieving their FP2020 goals to serve more women and provide a broader range of method options, including LARC methods.

Almost 26 million implants have been supplied to sub-Saharan African countries between 2013 and 2017, at a savings of over $230 million.

#### 5. High Impact Service Delivery Practices Have Led to Increased Implant Provision

Besides ensuring commodity cost reduction and greater availability, IAP partners and closely collaborating organizations made other investments also fundamentally necessary for quality, rights-based family planning service delivery. These include support for all-method counseling, training in proper implant insertion and removal technique, smooth functioning of supply chains, supportive supervision, local demand generation, and reliable client follow-up. In addition, a number of relevant high-impact service delivery practices (HIPs) being implemented more widely in family planning programs have helped increase equitable access to implant services. These include task shifting, community-based service provision, deployment of family planning-dedicated providers, and provision of family planning to hard-to-reach rural and peri-urban clients via mobile outreach services.^28^

Task shifting (or task sharing) of implants provision to lower-level cadres and frontline and community workers has been endorsed by WHO^29^ and proven to be effective in increasing implant access and use in various clinical and nonclinical settings. This has included provision of implants by community health extension workers (CHEWs) on a pilot basis in Nigeria^30^ and on a large scale in Ethiopia. In Ethiopia, more than 8,000 CHEWs were trained and subsequently provided implant services in the public sector to over 1.1 million women between 2009 and 2015.^31^ Use of vouchers to address inequities in access by poor and underserved groups including youth is also an emerging HIP that has led to increased implants provision and uptake.^32^ Use of vouchers in a social franchising program in Uganda in 2013-14 resulted in uptake of 165,000 implants (and 76,000 IUDs) in 24 months.^33^

Over 1 million women in Ethiopia have been provided an implant by a public-sector community health extension worker.

Aggregated service statistics further convey the extent, rapidity, and acceleration of recent rises in implant uptake. Between 2008 and 2012, MSI provided over 1.4 million implants to women in 10 of the 12 sub-Saharan African countries under review, with implant uptake rising almost 9-fold, from 73,000 in 2008 to over 600,000 in 2012.^34^ More than two-thirds of this service provision was delivered via mobile outreach and family planning-dedicated providers, mostly free of charge to clients. Subsequently, as implants have become more widely available, the number of women choosing an implant in MSI's 15 sub-Saharan African family planning programs has increased each year, from around 1 million in 2013, to 1.4 million in 2014, 1.7 million in 2015, 2.1 million in 2016, and 2.7 million in 2017, a 5-year total of almost 9 million implants provided to women during this time (Kathryn Church, written communication, January 2018). Of these clients, more than 50% were first-time or “lapsed” users of family planning (no use for the past 3 or more months), 38% were living in poverty (under $1.25/day), and around 12% were ages 15–19. Rapidly increasing uptake of implants has also occurred within the private provider networks affiliated with PSI, with more than 2.6 million implants provided in PSI's sub-Saharan African programs between 2013 and 2017, an increase of approximately 400% over the preceding 4 years (Pierre Moon, written communication, January 2018).

### Is the Uptake of Implants Likely to Continue?

The rapid uptake of implants highlighted in this article seems very likely to continue and perhaps even to accelerate in sub-Saharan Africa, as has been most notably the case in Kenya. Certainly the first order of sustainability—sustainability of client knowledge about, interest in, and experience with a method—is well on its way toward the tipping point of being firmly established in most of the 12 countries. Observations and factors that support this speculative prediction are categorized next, according to demand-side or supply-side considerations.

For many demand-side and supply-side reasons, the rapid uptake of implants is likely to continue, and even to accelerate.

#### Demand-Side Considerations

1. Global megatrends such as high rates of urban growth, greater women's education and participation in the formal workforce, and the spread of mass communication and social media will increasingly be driving normative change toward smaller desired family size and greater demand for contraception in every region of sub-Saharan Africa,^2^ as they have in other regions of the world.
2. The high current use of implants by women in most sociodemographic categories and high implant continuation rates imply likely activation of interpersonal and intra-community diffusion networks regarding women's (and men's) positive perceptions about implants. In turn, this may increase interest and use further, including more method-switching from shorter-acting methods that are discontinued more frequently, more demanding of user compliance, and may be less congruent with reproductive intentions.
3. Although married women without children generally have very low current use of implants—a reflection of low LARC demand and sociocultural pressures to have a first child and/or provider bias against offering the method to this category of women—they may well choose implants in the future, after giving birth. This is implied in the high current use of implants by married women of low parities (1–2 children) and by sexually active unmarried women.
4. Implants' suitability for women who wish to limit further childbearing is also important. As recently noted in this journal, the demand to limit is a widespread and rising reproductive intention in sub-Saharan Africa, even among younger women, and 37% of all demand for family planning among married women in sub-Saharan Africa is for limiting.^35^
5. Rising demand for implants has almost certainly also been occurring in other sub-Saharan African countries beyond the 12 countries included in this review. If more recent surveys in these other countries had been available, this likely could have been seen to be the case. In 2013–14 (DHS), Zambia already had attained an implant CPR of 5.5% and in 2014–15 (DHS) Rwanda had an implant CPR of 7.7%.
6. Although implant CPR has risen markedly, implant use is still well below that of injectables in 11 of the 12 countries. This too may imply greater future uptake of implants, as women currently using a progestin-only method of relatively short duration and higher circulating progestin levels (i.e., injectables) switch to a longer-acting progestin-only method that conveys lower circulating progestin levels and requires fewer routine interactions with the health system (i.e., implants).
7. Unmet need for family planning in sub-Saharan Africa, i.e., actual or latent demand, is currently the highest of any region of the world (21%).^36^ This could possibly get even larger in the future, given the health system challenges of meeting the needs of the burgeoning cohorts of youth entering their reproductive years,^16^ for whom implants are an appropriate option and likely to be appealing one when made more widely available to them.
8. The higher use of implants (and family planning) generated in urban settings suggests wider and greater uptake of implants nationally in future years, as cities are early-adopter harbingers of overall societal change. If this proves to be generally so in West Africa and Central Africa, examples from Niger and DRC hold promise. Whereas Niger's mCPR is only 14.4% and implant CPR is 1.7%, mCPR is 31.5% and implant CPR is 8.0% in its capital, Niamey (PMA2020 R3 2016). Similarly, mCPR in DRC several years ago was only 7.8% and implant CPR only 0.7%, whereas in Kinshasa mCPR was 19.0% and implant CPR was 2.4% (2013-14 DHS). These rose to 28.6% and 6.7% respectively by 2016 (PMA 2020 R5 2016). Increased uptake in parts of Nigeria has also prompted optimism about wider prospects for LARCs there.^37^

Cities are in the forefront of rising implant use, and sub-Saharan Africa is urbanizing rapidly.

Consistent with the above considerations, the Reproductive Health Supplies Coalition predicts demand for implants in the 69 IAP countries will rise steadily, from 13 million sets in 2016 to 25 million sets in 2022, totaling 125 million implant sets in the 7-year period from 2016 to 2022.^38^

#### Supply-Side, Service Policy, and Health System Considerations

1. Very importantly, as noted above, the IAP assurance of wide and substantial availability of Jadelle and Implanon NXT at the reduced price point has been extended until 2023. Levoplant is also being supplied more widely in sub-Saharan Africa at its comparable (slightly lower) commodity price point.
2. Diffusion of knowledge within provider and health system networks about the positive characteristics of implants is likely to increase, particularly as provider experience with implants' popularity and ease of insertion and removal increases, and as health systems increasingly recognize and seek to address the growing client demand. This likely will lead to more service providers, including frontline and community workers, offering implants as a method option more frequently and routinely, at more sites.
3. High-impact service delivery practices that enable wider access to implants, e.g., task shifting to frontline workers, are increasingly being endorsed by policy makers, even in regions where the practice has been limited previously, such as francophone West Africa.^39^ Interest in vouchers, social franchising, and private-sector provision of services is also growing, and these modalities are well suited to provision of implant (and other family planning) services, including to youth and other underserved groups with high unmet need and likely interest in implants.
4. Considerable time is required for internationally promulgated guidance to be adapted to national guidelines and local contexts, and then to diffuse into common health care practice, preservice professional education, and in-service training. This process is under way for implants and will undoubtedly occur increasingly over the next few years, including with respect to new and important guidance regarding suitability of implants for breastfeeding women, as well as for adolescents and young women, whether or not they have children.

Lowered implant commodity cost, of $6.90–$8.00 per set, is assured through at least 2023.

### What Challenges Need to Be Addressed?

In addition to the positive trends and opportunities discussed earlier, important health system, implant service, and cost/financing challenges need to be addressed now and increasingly in the future, to enable implant uptake to continue to rise.

#### Health System and Implant Service Considerations

Health system capacity to produce, train, employ, and deploy the large complements of health workers needed to make universal access to family planning a reality needs to be ensured. Implant removal services as well as insertion services must also be routinely and regularly available, accessible, and affordable.^40^^,^^41^ This can be daunting given the high volumes and principal modalities of service provision, e.g., mobile service provision in poor peri-urban and far-flung rural settings, especially when long intervals may have elapsed since the time of implant insertion. Capable management of the likely but unpredictable minor bleeding changes that implants cause must also be ensured, beginning with good counseling to explore how such changes might affect the client. New expert guidance that increases eligibility to use implants and new national guidelines based on this guidance do not automatically translate into new practices by providers comfortable with the status quo and perhaps uncomfortable providing contraception to young, unmarried, or nulliparous women. Rather, effecting such changes in provider practices requires time, knowledge transfer, and repeated program effort. Prompt availability of frequently conducted serial surveys has been valuable in documenting rapidly occurring changes in implant uptake and enabling the international family planning community to maintain focus on key program issues like implant removal and provision of equitable, rights-based services. Such surveys need to continue and to be undertaken in more countries.

Implant removal services must be routinely and regularly available, accessible, and affordable, on a wide scale.

Rapid serial surveys with prompt availability of findings have been very important to document progress and should be undertaken even more widely.

#### Cost and Financing Considerations

Although detailed analysis of cost and financing is beyond the purposes and scope of this article, it is clear these are aspects of paramount importance in ensuring sustainable implant service delivery programs. Even at the reduced access price point, the aggregate program cost of implant provision in sub-Saharan Africa over the next few years could easily exceed $500 million, especially if implant uptake in other countries approaches the arc of uptake seen in Kenya. Commodity cost alone in only the 12 countries included in this review exceeded $150 million. Furthermore, large, populous, and politically and economically important countries and regions like the DRC, francophone West Africa and Nigeria, currently with very low mCPR levels around 20% or lower, have only recent (and welcome) signs suggesting that more robust uptake of modern contraception including implants lies ahead there (and elsewhere). This will require even greater commitment and mobilization of resources from national and local governments, as well as from donors and service-providing partners, in order to meet growing demand and provide implant and other family planning services even more widely and equitably. Health insurance schemes and alternative funding models must also ensure that family planning is a universally covered, adequately reimbursed service.

There are also individual client-level cost considerations to be borne in mind. A very substantial proportion of the provision of implants documented in this article was delivered free of charge or at heavily subsidized rates to poor and disadvantaged clients by international NGOs. This almost certainly would not have happened without donor funding, which extended well beyond funding for commodity procurement. Reports from Senegal and elsewhere in West Africa attest to the disproportionally large lines of clients waiting for contraceptive services on “special free family planning days,”^42^ because services are not free on other days. Implant and other family planning services would undoubtedly drop off markedly if such approaches and funding were no longer available.

Free or highly subsidized services for disadvantaged and poor clients must be assured and/or expanded.

### What About the Prospects for the Other Reversible Modern Methods?

#### Injectables and Pills

For many African women, injectable contraceptives have been and remain the longest-acting and most effective modern method they can easily access. Injectables are also more convenient for many women than shorter-acting resupply methods, requiring “only” 4 routine client actions per year rather than, say, the pill's 365-plus. Injectables are also relatively easy for health systems to provide. These factors likely contribute to the injectable's substantial market share in most of the countries under review and elsewhere, and perhaps to the pill's substantially lower use. Injectables could be embarked on a similar trajectory as pills, however, upon greater availability and accessibility of a longer-acting, more “user-friendly” method (the implant), one with immediate rather than delayed return to fertility. If so, the proportionate declines in injectable use that have occurred in 9 of the countries could become absolute declines more widely. A countervailing dynamic toward greater injectable use, however, is likely to be the widening programmatic availability of the subcutaneous injectable, Sayana Press, with its prospects of enabling wider community-based provision as well as home-based provision and self-injection.^43^

The high prevalence of injectable use in sub-Saharan Africa is likely to decline as implant access and use continue to rise.

#### IUDs

Although use of the (copper-containing) IUD has been very low in almost all sub-Saharan African countries for many years, and it has been beset by myths and rumors among providers and clients alike, this might not necessarily be the case in the future. Hopeful signs are the modest increases in IUD use registered in 11 of the 12 countries. If investments in expanded LARC availability and service delivery continue, IUD use may continue to rise. In such efforts between 2013 and mid-2017, provider networks affiliated with PSI provided over 2 million IUDs in 12 sub-Saharan African countries (Pierre Moon, written communication, January 2018), 7 of which are included in this report's analysis. There is also increasing interest in the hormonal IUD (the levonorgestrel-releasing intrauterine system) in the international family planning community. Hormonal IUD use is rising in many industrialized countries including the United States, where IUD prevalence has risen to 6%.^44^ The hormonal IUD is in a similar situation to that of the implant several years ago: many positive method characteristics but too costly for routine and widespread programmatic use. Recent calls have been made for increased donor and program attention to this method's potential prospects,^45^^,^^46^ if commodity cost can be reduced.^47^

Modest gains in IUD use may continue.

## CONCLUSION

Implant availability, access, and use have risen substantially, very rapidly, and fairly equitably, at rates not often seen in family planning programs. This is a major, ongoing family planning success story. A hitherto largely unavailable contraceptive method is now being accessed widely by women across almost all sociodemographic categories in many sub-Saharan African countries. In a range of culturally varied, geographically widespread, economically disparate, and programmatically diverse country contexts, use of implants now accounts for one-fourth to one-half of all use of modern contraception. Method choice has been expanded, with implants becoming the most widely used method in Burkina Faso and the second most widely used method in 9 other countries. Increased implant use has been the main driver of the increased contraceptive use attained the past several years by 11 of the 12 sub-Saharan African countries analyzed in this article. With continued government and program commitment, mobilization of domestic resources, donor support, and private-sector engagement, these trends are likely to continue for at least the next few years. Important cost and service system challenges loom, however, if implant access is to be maintained and enlarged, in keeping with projected increases in demand.

The rapid, substantial, and generally equitable increases in implant access and use represent a major, ongoing family planning success story.

**ADDENDUM:** After the December 31, 2017, cutoff for inclusion in this study, the trends of rapid and substantial increase in implant use, share of method mix, and contribution to overall gains in mCPR have continued, as shown in the Appendix Table for the 3 PMA2020 Family Planning Briefs posted online in January 2018. Niger's implant prevalence increased from 1.7% in 2016 to 3.1% in 2017. This annual gain of 1.4 percentage points exceeds the high recent annual gains of 4 of the 7 countries shown in Table 4. Implant prevalence in DRC/Kinshasa is now 10.1%, higher than 9 countries included in this article and representing 37.9% of the current modern method mix. This substantial annual gain of 3.4 percentage points in implant prevalence exceeds DRC/Kinshasa's noteworthy annual gain in mCPR of 3.3 percentage points. Use of implants by sexually active unmarried women in DRC/Kinshasa also rose, from an implant CPR of 3.5% to 5.2%—13% of all their modern method use. The leapfrogging pattern is also seen in Ghana, where implant prevalence is now 8.4%, representing 30.7% of the current modern method mix—a level higher than 8 of the countries included in the analysis. Ghana's annual gain in implant CPR of 2.5 percentage points is 56% higher than its solid annual gain in mCPR of 1.6 percentage points. Implants have now become the most widely used modern method in DRC/Kinshasa and Ghana.

## Appendix

**APPENDIX TABLE.** Rapid and Substantial Gains in Implant Use, Share of Method Mix, and Contribution to mCPR Gains, Married Women, All Countries With PMA2020 Family Planning Briefs Posted Online in January and February 2018

Column 1	Column 2	Column 3	Column 4	Column 5	Column 6	Column 7	Column 8
Country and Data Source	mCPR (%)	Implant CPR (%)	Implant Share of Method Mix (%)	Injectable CPR (%)	Injectable Share of Method Mix (%)	mCPR Annual Gain, 2016 to 2017 (pp)	Implant CPR Annual Gain, 2016 to 2017 (pp)
DRC/K PMA R6 2017	26.7	**10.1**	**37.9**	5.2^a^	19.5^a^	**3.3**	**3.4**
DRC/K PMA R5 2016	23.4	**6.7**	**28.6**	3.1^a^	13.3^a^		
Ghana PMA R6 2017	27.4	**8.4**	**30.7**	7.8	28.5	**1.6**	**2.5**
Ghana PMA R5 2016	25.8	**5.9**	**23.0**	8.4	32.6		
Niger PMA R2 2017	18.1	**3.1**	**17.1**	7.3	40.3^a^	**3.7**	**1.4**
Niger PMA R1 2016	14.4	**1.7**	**11.9**	5.0	34.9		

Abbreviations: CPR, contraceptive prevalence rate; DMPA, depot medroxyprogesterone acetate; DRC/K, Democratic Republic of the Congo/Kinshasa only; mCPR, modern contraceptive prevalence rate; PMA, Performance Monitoring and Accountability 2020; pp, percentage point; R, round.

a Sum of the intramuscular DMPA injectable and the subcutaneous injectable Sayana Press.

## Notes

**Peer Reviewed**

**First Published Online:** March 20, 2018

**Funding:** None.

## References

[1] United Nations (UN), Department of Economic and Social Affairs, Population Division. World Contraceptive Patterns 2013. New York: UN; 2013. http://www.un.org/en/development/desa/population/publications/family/contraceptive-wallchart-2013. Accessed February 27, 2018.

[2] World Development Indicators. Washington, DC: World Bank. http://wdi.worldbank.org/table/2.1. Accessed October 20, 2017.

[3] Jacobstein R Stanley H. Contraceptive implants: providing better choice to meet growing family planning demand. Glob Health Sci Pract. 2013;1(1):11–17. 10.9745/GHSP-D-12-00003. 25276512 PMC4168562

[4] DHS Overview. The DHS Program website. https://dhsprogram.com/What-We-Do/Survey-Types/DHS.cfm. Accessed February 27, 2018.

[5] PMA2020/Publications/Briefs/Family Planning. Performance Monitoring and Accountability 2020 (PMA2020) website. http://pma2020.org/fp-briefs. Accessed February 27, 2018.

[6] Tumlinison K Okigpo C Speizer I. Provider barriers to family planning access in urban Kenya. Contraception. 2015;92(2): 143–151. 10.1016/j.contraception.2015.04.002. 25869629 PMC4506861

[7] Sidze EM Lardoux S Speizer IS Faye CM Mutua MM Badji F. Young women's access to and use of contraception: the role of providers' restrictions in urban Senegal. Int Perspect Sex Reprod Health. 2014;40(4):176–184. 10.1363/4017614. 25565345 PMC6652199

[8] Ross J Stover J. Developing a family planning goal for 2015 that supports MDG-5b. Washington, DC: Health Policy Initiative, Task Order I; 2010. http://www.healthpolicyplus.com/archive/ns/pubs/hpi/1310_1_FP_Goal_for_2015_FINAL_acc.pdf. Accessed February 27, 2018.

[9] Salem RM Jacobstein R Yacobson I Spieler J Frazee E. Essential knowledge about hormonal implants. Implants Toolkit website. 2010. https://www.k4health.org/toolkits/implants/essential-knowledge-about-hormonal-implants. Accessed February 21, 2018.

[10] Ali M Bahamondes L Bent Landoulsi S. Extended effectiveness of the etonogestrel-releasing contraceptive implant and the 20 µg levonorgestrel-releasing intrauterine system for 2 years beyond U.S. Food and Drug Administration product labeling. Glob Health Sci Pract. 2017;5(4):534–539. 10.9745/GHSP-D-17-00296. 29263025 PMC5752601

[11] U.S. Centers for Disease Control and Prevention. Effectiveness of Family Planning Methods. Atlanta, GA: CDC. https://www.cdc.gov/reproductivehealth/unintendedpregnancy/pdf/contraceptive_methods_508.pdf. Accessed January 12, 2018.

[12] Jacobstein R Polis C. Progestin-only contraception: injectables and implants. Best Pract Res Clin Obstet Gynaecol. 2014;28(6):795–806. 10.1016/j.bpobgyn.2014.05.003. 24996766

[13] World Health Organization (WHO). Medical Eligibility for Contraceptive Use. 5th edition. Geneva: WHO; 2015. http://www.who.int/reproductivehealth/publications/family_planning/MEC-5/en/. Accessed February 27, 2018.

[14] Global Consensus Statement for Expanding Contraceptive Choice for Adolescents and Youth to Include Long-Acting Reversible Contraception. FP2020 website. http://www.familyplanning2020.org/youth-larc-statement. Accessed February 27, 2018.

[15] Committee on Adolescent Health Care Long-Acting Reversible Contraception Working Group, The American College of Obstetricians and Gynecologists. Committee opinion no. 539: adolescents and long-acting reversible contraception: implants and intrauterine devices. Obstet Gynecol. 2012;120(4):983–988. 10.1097/AOG.0b013e3182723b7d. 22996129

[16] About Us. Family Planning 2020 (FP2020) website. http://www.familyplanning2020.org/microsite/about-us. Accessed February 27, 2018.

[17] Family Planning 2020 (FP2020). *FP2020**:* The Way Ahead, 2016–2017. Washington, DC: FP2020; 2017. http://progress.familyplanning2020.org/en. Accessed February 27, 2018.

[18] Ministry of Health (MOH) [Ethiopia]. Costed Implementation Plan for Family Planning in Ethiopia, 2015/16–2020. Addis Ababa, Ethiopia: MOH; 2016. http://www.healthpolicyplus.com/ns/pubs/2021-2030_EthiopiaCIPNov.pdf. Accessed February 27, 2018.

[19] Ministry of Health (MOH) [Uganda]. Uganda Family Planning Costed Implementation Plan, 2015–2020. Kampala, Uganda: MOH; 2014. https://www.healthpolicyproject.com/ns/docs/CIP_Uganda.pdf. Accessed February 27, 2018.

[20] Global - Summary of Shipments. UNFPA Procurement Services website. https://www.unfpaprocurement.org/rhi-home. Accessed February 27, 2018.

[21] Clinton Health Access Initiative (CHAI). Case study: expanding global access to contraceptive implants. Boston, MA: CHAI; 2015. https://clintonhealthaccess.org/content/uploads/2015/08/Case-Study_LARC.pdf. Accessed February 27, 2018.

[22] Implant Access Program. Implant Access Program: expanding family planning options for women. 2016. http://www.familyplanning2020.org/resources/13386. Accessed February 27, 2018.

[23] Bayer. Bayer halves the price of its contraceptive implant Jadelle® for women in developing countries. 2016. http://www.familyplanning2020.org/articles/12388. Accessed February 27, 2018.

[24] Family Planning 2020 (FP2020). 2016 FP2020 annual commitment update questionnaire response: Merck (MSD). http://ec2-54-210-230-186.compute-1.amazonaws.com/wp-content/uploads/2016/09/FP2020_2016_Annual_Commitment_Update_Questionnaire-Merck_DLC.pdf. Accessed February 27, 2018.

[25] FHI 360; Shanghai Dahua Pharmaceutical Co., Ltd. Sino-implant (II)/Levoplant overview. Durham, NC: FHI 360; 2017. https://www.fhi360.org/sites/default/files/media/documents/resource-sino-levoplant-overview.pdf. Accessed February 27, 2018.

[26] DKT International and Shanghai Dahua Pharmaceutical partner to increase access to contraceptive implants: price of Levoplant reduced to $6.90 in FP2020 countries [news release]. Washington, DC: DKT International; February 13, 2018. https://2umya83uy24b2nu5ug2708w5-wpengine.netdna-ssl.com/wp-content/uploads/2018/02/Global-Levoplant-Partnership_PR.pdf. Accessed February 27, 2018.

[27] Global - Quantity Summary. UNFPA Procurement Services website. https://www.unfpaprocurement.org/rhi-home. Accessed February 27, 2018.

[28] High Impact Practices in Family Planning (HIPs). Family planning high impact practices list. Washington, DC: United States Agency for International Development; 2018. http://fphighimpactpractices.org/high-impact-practices-in-family-planning-list-2/. Accessed February 27, 2018.

[29] World Health Organization (WHO), Department of Reproductive Health and Research. Task shifting to improve access to contraceptive methods. Geneva: WHO; 2013. http://apps.who.int/iris/bitstream/10665/94831/1/WHO_RHR_13.20_eng.pdf?ua=1. Accessed February 27, 2018.

[30] Charyeva Z Oguntunde O Orobaton N. Task shifting provision of contraceptive implants to community health extension workers: results of operations research in northern Nigeria. Glob Health Sci Pract. 2015;3(3):382–394. 10.9745/GHSP-D-15-00129. 26374800 PMC4570013

[31] Tilahun Y Lew C Belayihun B Lulu Hagos K Asnake M. Improving contraceptive access, use, and method mix by task sharing Implanon insertion to frontline health workers: the experience of the Integrated Family Health Program in Ethiopia. Glob Health Sci Pract. 2017; 5(4):592–602. 10.9745/GHSP-D-17-00215. 29229650 PMC5752606

[32] Menotti EP Farrell M. Vouchers: a hot ticket for reaching the poor and other special groups with voluntary family planning services. Glob Health Sci Pract. 2016;4(3):384–393. 10.9745/GHSP-D-16-00084. 27669707 PMC5042695

[33] Bellows B Mackay A Dingle A Tuyiragize R Nnyombi W Dasgupta A. Increasing contraceptive access for hard-to-reach populations with vouchers and social franchising in Uganda. Glob Health Sci Pract. 2017;5(3):446–455. 10.9745/GHSP-D-17-00065. 28963175 PMC5620340

[34] Duvall S Thurston S Weinberger M Nuccio O Fuchs-Montgomery N. Scaling up delivery of contraceptive implants in sub-Saharan Africa: operational experiences of Marie Stopes International. Glob Health Sci Pract. 2014;2(1):72–92. 10.9745/GHSP-D-13-00116. 25276564 PMC4168608

[35] Van Lith LM Yahner M Bakamjian L. Women's growing desire to limit births in sub-Saharan Africa: meeting the challenge. Glob Health Sci Pract. 2013;1(1):97–107. 10.9745/GHSP-D-12-00036. 25276520 PMC4168554

[36] Guttmacher Institute. Adding It Up: Investing in Contraception and Maternal and Newborn Health in Africa. New York: Guttmacher Institute; 2017. https://www.guttmacher.org/fact-sheet/adding-it-up-contraception-mnh-2017-africa. Accessed February 27, 2018.

[37] Shelton JD Finkle C. Leading with LARCs in Nigeria: the stars are aligned to expand effective family planning services decisively. Glob Health Sci Pract. 2016;4(2):179–185. 10.9745/GHSP-D-16-00135. 27353612 PMC4982243

[38] Reproductive Health Supplies Coalition. Summary of April 2016 CSP Contraceptive Implant Demand Forecast. Brussels, Belgium: Reproductive Health Supplies Coalition; 2016. https://www.rhsupplies.org/uploads/tx_rhscpublications/Summary_of_August_2016_CSP_Contraceptive_Implant_Demand_Forecast.pdf. Accessed February 27, 2018.

[39] Family Planning Ouagadougou Partnership. 3rd Annual Ouagadougou Partnership Meeting: Paris, France, December 18–19, 2014. Dakar, Senegal: IntraHealth International, Bureau du Sénégal; 2015. http://ec2-54-210-230-186.compute-1.amazonaws.com/wp-content/uploads/2015/03/Report_OPCU-Paris-Meeting_ENG_final.pdf. Accessed February 27, 2018.

[40] Sergison JE Stalter RM Callahan RL Rademacher KH Steiner MJ. Cost of contraceptive implant removal services must be considered when responding to the growing demand for removals. Glob Health Sci Pract. 2017;5(2):330–332. 10.9745/GHSP-D-17-00100. 28655806 PMC5487094

[41] Christofield M Lacoste M Accessible contraceptive implant removal services: an essential element of quality service delivery and scale-up. Glob Health Sci Pract. 2016;4(3):366–372. 10.9745/GHSP-D-16-00096. 27577239 PMC5042693

[42] The Challenge Initiative. Boîte a outils du paquet d'interventions à haut impact sur la planification familiale. http://www.aimf.asso.fr/IMG/pdf/brochure_boite_a_outils_du_paquet_porteur_tci_afrique_ouest_annexe_3.pdf. Accessed February 27, 2018.

[43] PATH. How to Introduce and Scale Up Sayana Press (DMPA-SC in Uniject): Practical Guidance from PATH Based on Lessons Learned During Pilot Introduction. Seattle, WA: PATH; 2017. http://www.path.org/publications/files/RH_sp_dmpa_sc_practical_guidance_2017.pdf. Accessed February 27, 2018.

[44] Branum AM Jones J. Trends in long-acting reversible contraception use among U.S. women aged 15-44. NCHS Data Brief No. 188. Hyattsville (MD): U.S. Department of Health and Human Services, Centers for Disease Control and Prevention, National Center for Health Statistics; 2015. http://www.cdc.gov/nchs/data/databriefs/db188.htm. Accessed February 27, 2018.

[45] Hubacher D. The levonorgestrel intrauterine system: reasons to expand access to the public sector of Africa. Glob Health Sci Pract. 2015;3(4): 532–537. 10.9745/GHSP-D-15-00178. 26681701 PMC4682579

[46] Jacobstein R Shelton JD. The levonorgestrel intrauterine system: a pragmatic view of an excellent contraceptive. Glob Health Sci Pract. 2015;3(4):538–543. 10.9745/GHSP-D-15-00330. 26681702 PMC4682580

[47] Rademacher KH Solomon M Brett T. Expanding access to a new, more affordable levonorgestrel intrauterine system in Kenya: service delivery costs compared with other contraceptive methods and perspectives of key opinion leaders. Glob Health Sci Pract. 2016;4(suppl 2):S83–S93. 10.9745/GHSP-D-15-00327. 27540128 PMC4990165

